# Cellular Responses During Kidney Normothermic Machine Perfusion Reflect Graft Outcomes

**DOI:** 10.1016/j.ekir.2025.08.017

**Published:** 2025-08-20

**Authors:** Shengbing Li, Hector Tejeda-Mora, Julia S. Slagter, Daphne M. Hullegie-Peelen, Iacopo Cristoferi, Yitian Fang, Sarah Bouari, David Schumacher, Anne Babler, Felix Schreibing, Teresa Anslinger, Marlies E.J. Reinders, Rafael Kramann, Robert C. Minnee, Martin J. Hoogduijn

**Affiliations:** 1 Division of Nephrology and Transplantation, Department of Internal Medicine, Erasmus MC Transplant Institute, University Medical Center Rotterdam, The Netherlands; 2 Division of HPB and Transplant Surgery, Department of Surgery, Erasmus MC Transplant Institute, University Medical Center Rotterdam, The Netherlands; 3 Department of Anesthesiology, Medical Faculty, RWTH Aachen University, Aachen, Germany; 4 Department of Medicine 2 (Nephrology, Immunology, Rheumatology, Hypertension), Medical Faculty, RWTH Aachen University, Aachen, Germany

**Keywords:** acute rejection, kidney transplantation, normothermic machine perfusion, quantitative real-time PCR, single nucleus RNA sequencing

## Abstract

**Introduction:**

Kidneys donated after circulatory death and kidneys from older donors demonstrate increased susceptibility to ischemia reperfusion injury and immune attack after transplantation. However, there are currently no assessment tools that can reliably predict posttransplant outcome of donor kidneys. In this study, we investigated whether the cellular responses of kidneys during normothermic machine perfusion (NMP) are associated with posttransplant outcome.

**Methods:**

Snap-frozen biopsies from 6 kidneys taken before and after 2-hour NMP were subjected to single nucleus RNA sequencing (snRNA seq). Biopsies from another 22 kidneys taken at the same time points were included for quantitative real-time PCR (qPCR) to confirm the snRNA seq data. Estimated glomerular filtration rates (eGFRs) until 30 days posttransplant were used to determine posttransplant function.

**Results:**

snRNA seq analyses of kidney biopsies identified 38,451 cells distributed across 11 distinct cell types. NMP induced an upregulation of genes for adenosine triphosphate (ATP) production–related proteins, heat shock proteins (HSPs), transporter proteins, and proteins that prevent protein misfolding. These findings were confirmed by the qPCR analyses. We observed no significant gene differences between delayed graft function (DGF) and non-DGF kidneys. However, after NMP, the expression of HSP was above average in 3 kidneys that experienced acute rejection in the first 10 days after transplantation compared with nonrejecting kidneys.

**Conclusion:**

This study demonstrates that 2-hour NMP impacts gene expression profiles of the majority of cell types of the kidney. It suggests that NMP triggers differential gene expression patterns in kidneys with an increased risk for early rejection.

## Graphical abstract

**Figure:**
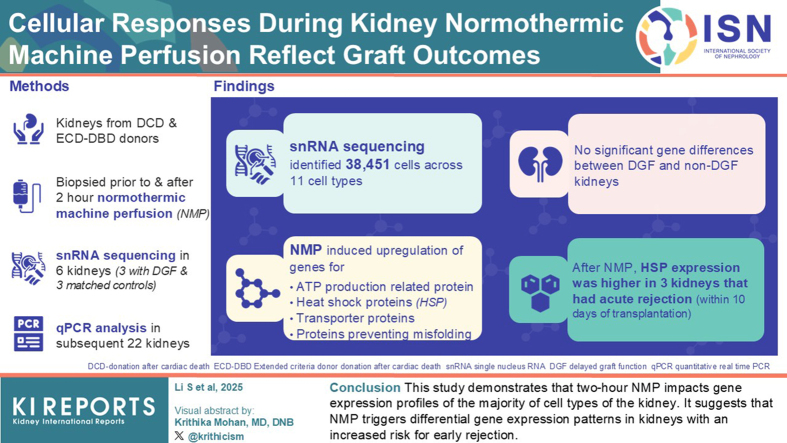

Kidney transplantation is a monumental achievement of 20th-century medicine and has remarkably amplified the lifespans of thousands of patients with end-stage kidney disease worldwide. Nevertheless, the shortage of appropriate donor kidneys is leading to a growing number of recipients on the waiting list. To increase the donor pool, acceptance criteria for donation are extended. Donation after circulatory death (DCD) and donation after brain death (DBD) kidneys from older donors or donors with cardiovascular comorbidities, so called extended criteria DBD (ECD-DBD) donors, are thus accepted in order to increase the number of transplanted patients. However, these kidneys demonstrate increased susceptibility to ischemia reperfusion injury and immunogenic responses.^1^ Therefore, it is imperative to assess the function of high-risk kidneys; however, there are no tools to empirically assess their quality prior to transplantation.

Optimal preservation strategies are of utmost importance to minimize damage of high-risk organs outside the body. Commonly used techniques for organ preservation are static cold storage and hypothermic machine perfusion (HMP).^2^ These techniques aim to decrease metabolic activity, thereby effectively minimizing ischemic damage. However, in a cold preservation environment, the opportunity for functional assessment of the kidneys is precluded, because metabolism is reduced to a minimum.

To allow donor organs become metabolically active, NMP was developed.^3^ It combines cardiopulmonary bypass technology and extracorporeal membrane oxygenation, which enables an oxygen-rich perfusate to circulate through the kidney at normal body temperature and physiological arterial pressure.^4^ Promisingly, it has been suggested that NMP may restore donor kidney function *ex vivo*,^5^ and it has been demonstrated that NMP supports cellular metabolism and replenishes ATP levels.^6^^,^^7^ A recent study demonstrated that it is feasible to apply NMP to donor kidneys in the clinical setting, and although 1-hour NMP did not improve posttransplant outcome, it shows that NMP offers a window for assessment of donor kidneys.^8^

This view is corroborated by the surge in renal hormone release during NMP as demonstrated in our previous study.^9^ Thus, NMP may allow functional evaluation of fully metabolically active kidneys.^10^

Currently, the measurements that are used to assess function during NMP are restricted to renal blood flow, renal resistance, and metabolic parameters such as pH and acid-base balance.^11^ A global transcriptional profiling of NMP kidneys has demonstrated that NMP upregulated inflammatory gene expressions. The addition of hemoadsorber to the NMP circuit can reduce those cytokine levels.^12^ Although these findings suggest that removing proinflammatory mediators during NMP may improve organ viability, it is yet to be determined whether these responses can prognosticate posttransplantation outcomes. Furthermore, because the kidney contains diverse cell types, the response of individual cell types to NMP remains unclear. Thus, understanding molecular responses to NMP is crucial to learn about the potential and limitation of this technology.

Here, we approached these questions by performing transcriptomic analysis of human kidney biopsies taken before and after 2-hour NMP to assess global changes in gene expression. We also explored whether gene expression changes during NMP can serve as a relevant indicator for kidney functionality posttransplantation in DCD and ECD-DBD kidneys.

## Methods

### Patient Population

Kidneys from DCD and ECD-DBD donors were subjected to 2-hour NMP. The first 3 kidneys with DGF in the cohort were selected for snRNA seq analysis and 3 kidneys that were matched for key clinical variables such as male, DCD, and of similar age, were selected as controls. Subsequent 22 kidneys were used for qPCR analysis.

Donor or recipient characteristics, parameters during NMP, and clinical outcomes in recipients within 1 month of transplantation were recorded (Table 1, Table 2, Table 3, Table 4). All recipients received basiliximab as induction therapy. Hourly urine production was collected for verifying stable NMP operation, and eGFR and slopes of eGFR recovery via linear regression in the first month after transplantation were used to assess early graft function. In our dataset, eGFR was set to 0 for all patients with no measurable kidney function at a given time point, including those who did not meet formal DGF criteria. This approach captures early functional impairment. In addition, needle biopsies from 2 kidneys that were not suitable for transplantation because of prolonged ischemia and acute kidney injury, but that underwent 2-hour NMP were collected to serve as examples of poor-quality kidneys.

**Table 1:** Donor characteristics

Kidney no.	Donor type	Donor gender	Donor age (yr)	Donor WIT (min)	Donor weight (kg)	Donor BMI	Donor hypertension	Donor diabetes	Donor creatine (μmol/l)	Cause of death	Analysis method
K1	DCD	M	29	11	85	28	-	0	94	Intra cerebral bleeding	snRNA seq
K2	DCD	M	65	13	95	27	3	0	48	Postanoxic encaphalopathy after OHCA	snRNA seq
K3	DCD	M	72	16	83	26	0	0	59	Ischemic CVA	snRNA seq
K4	DCD	M	36	25	70	21.6	0	0	49	Suicide	snRNA seq
K5	DCD	M	71	14	95	31	0	2	71	Postanoxic encephalopathy after OHCA	snRNA seq
K6	DCD	M	57	11	98	29	4	1	89	Postanoxic encephalopathy after OHCA	snRNA seq
K7	DCD	M	49	14	90	27	0	0	138	Postanoxic encephalopathy after OHCA	qPCR
K8	DCD	F	72	13	74	25.6	0	0	55	Trauma capitis	qPCR
K9	ECD-DBD	M	69	0	90	28	0	0	94	Cerebral bleeding	qPCR
K10	ECD-DBD	F	70	0	54	20	0	0	43	Trauma capitis	qPCR
K11	DCD	F	60	27	65	22	3	0	104	Ischemic CVA	qPCR
K12	DCD	F	49	15	80	30	0	0	80	Postanoxic encephalopathy after OHCA	qPCR
K13	DCD	M	67	16	98	28	4	0	108	Cerebral bleeding	qPCR
K14	DCD	F	74	17	92	34	0	0	71	Postanoxic encephalopathy after OHCA	qPCR
K15	DCD	M	63	25	83	25	0	0	53	Trauma capitis	qPCR
K16	ECD-DBD	F	65	0	100	32	1	0	40	Cerebral bleeding	qPCR
K17	DCD	F	59	15	75	24	1	0	47	Cerebral bleeding	qPCR
K18	DCD	M	63	26	76	27	0	0	64	Trauma capitis	qPCR
K19	DCD	F	66	38	73	27	0	0	54	Cerebral bleeding	qPCR
K20	DCD	M	65	18	80	26	1	0	99	Euthanasia	qPCR
K21	DCD	M	70	25	80	25	0	0	97	Cerebral bleeding	qPCR
K22	DCD	M	46	22	92	30	0	0	94	Euthanasia	qPCR
K23	ECD-DBD	M	77	0	70	24	0	0	77	Trauma capitis	qPCR
K24	DCD	M	27	15	70	20	0	0	101	Trauma capitis	qPCR
K25	DCD	M	79	20	95	29	3	0	60	Postanoxic encephalopathy after OHCA	qPCR
K26	ECD-DBD	F	75	0	62	22	1	0	46	Cerebral bleeding	qPCR
K27	DCD	M	66	12	89	29	0	0	62	Trauma capitis	qPCR
K28	ECD-DBD	M	64	0	80	27	0	0	55	Cerebral bleeding	qPCR
KB1	DCD	M	47	24	74	27	0	0	140	OCHA based on VF	qPCR
KB2	DCD	M	70	18	88	25	0	0	85	Cardiac arrest	qPCR

BMI, body mass index; CVA, cerebral vascular accident; DCD, donation after circulatory death; donor WIT, warm ischemia time in the donor after the cessation of life support and before the cold flush in the aorta; ECD-DBD, extended criteria donation after brain death; F, female; M, male; OCHA, out-of-hospital cardiac arrest; qPCR, quantitative real-time polymerase chain reaction; snRNA seq, single nucleus RNA sequencing; VF, ventricular fibrillation.

In donor hypertension and diabetes, 0 is considered to be donors with no history of hypertension/diabetes, 1 is considered to be donors with a 0–5 yrs history of hypertension/diabetes, 2 is considered to be donors with a 6–10 yrs history of hypertension/diabetes, and 3 is considered to be donors with > 10 yrs history of hypertension/diabetes; “-” means missed data.

**Table 2:** Characteristics of normothermic machine perfusion

Kidney no.	CIT1 (h)	CIT2 (h)	Total CIT (h)	Urine output 60 min (ml/h)	Urine output 120 min (ml/h)
K1	6.25	0.87	7.12	96	90
K2	13.25	1.08	14.33	0	1.7
K3	12.78	0.52	13.30	210	185
K4	4.53	2.85	7.38	221	230
K5	6.62	2.70	9.32	13	65
K6	9.73	1.43	11.16	22	71
K7	7.43	2.27	9.70	28	52
K8	8.32	3.48	11.80	56	68
K9	11.15	1.17	12.32	105	84
K10	6.30	1.03	7.33	102	166
K11	6.72	1.93	8.65	78	152
K12	7.10	0.50	7.60	209	324
K13	5.30	6.72	12.02	17	29
K14	8.65	2.50	11.15	105	95
K15	10.08	0.97	11.05	72	245
K16	6.05	1.75	7.80	72	115
K17	5.57	0.85	6.42	38	56
K18	10.40	1.10	11.50	38	29
K19	6.72	5.47	12.19	304	339
K20	6.85	0.58	7.43	118	130
K21	7.82	1.95	9.77	90	30
K22	5.12	1.42	6.54	15	100
K23	5.88	3.63	9.51	52	62
K24	5.30	1.02	6.32	270	150
K25	6.97	4.82	11.79	42	47
K26	11.63	1.33	12.96	130	435
K27	10.25	0.78	11.03	25	35
K28	6.43	2.33	8.76	94	125
KB1	25.40	-	25.40	13	17
KB2	12.70	-	12.70	43	53

CIT-1, cold ischemia time before normothermic machine perfusion; CIT-2, cold ischemia time on static cold storage after disconnecting from the normothermic machine perfusion device until transplantation.

KB1 and KB2 as discarded kidneys do not have CIT2.

**Table 3:** Transplantation characteristics

Kidney no.	DGF	DGF duration (days)	PNF	Acute rejection	Acute rejection day	Rejection type & Banff diagnosis	Banff v-lesions (arteritis)	eGFR day 0	eGFR day 7	eGFR day 14	eGFR day 30
K1	Yes	6	No	No				0	7	12	27
K2	Yes		Yes	Yes	8	aTCMR2a	yes	0	0	0	0
K3	No		No	No				8	16	22	15
K4	No		No	No				12	67	65	58
K5	No		No	No				4	5	6	31
K6	Yes	4	No	Yes	8	aTCMR2a	yes	0	9	17	38
K7	Yes	7	No	No				0	0	22	64
K8	No		No	No				12	41	43	44
K9	Yes	4	No	No				0	10	14	14
K10	Yes	5	No	No				0	22	34	47
K11	No		No	Yes	10	aTCMR2b	yes	0	12	15	12
K12	No		No	No				3	16	29	37
K13	Yes	-	Yes	Yes	9	aTCMR3	yes	0	0	0	0
K14	No		No	No				20	34	46	56
K15	No		No	No	90	aTCMR2a	yes	7	8	16	34
K16	Yes	7	No	No				0	0	14	29
K17	Yes	9	No	No				0	0	27	31
K18	No		No	Yes	127	aTCMR2b	yes	6	23	65	45
K19	No		No	No				6	11	24	32
K20	No		No	No				6	29	43	42
K21	Yes	32	No	No				0	0	0	0
K22	No		No	No				6	16	30	39
K23	Yes	73	No	No				0	0	0	0
K24	No		No	No				6	48	71	73
K25	Yes	37	No	No				0	0	0	0
K26	No		No	No				6	40	16	34
K27	Yes	27	No	No	152	aTCMR1a	no	0	0	0	11
K28	No		No	No				14	34	47	65

aTCMR, acute T-cell–mediated rejection; DGF, delayed graft function, defined as the need for dialysis in the first week after transplantation; eGFR, estimated glomerular filtration rate, expressed as (mL/min per 1.73 m^2^); PNF, primary nonfunction, defined as a persistent need for dialysis 3 months after transplantation.

Zero eGFR indicates the patient is on dialysis.

**Table 4:** Recipient characteristics

Kidney no.	Age (yr)	Gender	Residual kidney function (creatine μmol/l)	Re-transplantation status	vPRA	HLA mismatch	UPCR day 7 (g/mol)	UPCR day 14 (g/mol)	UPCR day 30 (g/mol)
K1	67	M	866	0	0	1.1.1	100	16	11
K2	77	M	518	0	0	2.2.2	76	34	29
K3	72	F	467	0	24	1.2.2	75	63	35
K4	67	M	799	0	22	2.1.0	51	104	45
K5	71	M	1176	0	0	1.1.2	36	20	18
K6	60	M	499	0	0	1.1.1	171	159	135
K7	67	M	323	0	0	0.2.0	63	11	29
K8	74	F	466	0	0	2.1.2	45	38	28
K9	69	F	459	0	0	0.1.0	55	17	12
K10	71	M	562	0	0	1.1.2	219	47	19
K11	68	M	588	0	0	0.1.1	83	35	39
K12	31	M	1506	0	0	1.1.2	80	26	20
K13	79	F	360	0	6	2.2.2	584	513	121
K14	69	F	192	0	0	1.2.1	68	33	39
K15	63	M	667	0	80	1.2.2	50	N/A	30
K16	72	M	558	0	0	2.2.2	69	75	150
K17	50	F	404	1	0	0.1.1	152	142	111
K18	63	F	891	0	8	1.1.0	114	N/A	56
K19	66	M	800	0	0	0.2.1	53	30	36
K20	72	F	589	0	69	2.2.2	84	29	21
K21	79	M	776	0	0	0.1.1	3759	58	69
K22	58	M	819	1	0	1.1.0	103	21	12
K23	78	M	465	0	0	1.2.2	67	113	44
K24	61	M	763	0	0	0.1.2	19	22	14
K25	75	M	506	0	0	2.2.2	58	47	47
K26	67	M	736	0	0	1.2.2	41	11	18
K27	67	F	462	0	22	2.2.2	37	44	21
K28	25	M	937	0	0	1.1.1	61	68	52

F, female; HLA, human leukocyte antigen; M, male; N/A, not applicable; UPCR, urine protein creatinine ratio; vPRA, virtual panel-reactive antibodies.

Retransplantation status: 0 means no retransplantation, 1 means 1 previous kidney transplant.

### NMP Execution

The kidneys included in this study were part of an ongoing randomized controlled trial, registered under the identifier NCT04882254 on clinicaltrials.gov in which donor kidneys were randomly assigned to either the HMP control or NMP intervention group.^13^ Donor kidneys were transported on pulsatile HMP after procurement. Moreover, based on the findings from the COMPARE trial,^14^ kidneys from donors aged ≥ 50 years were treated with oxygenated HMP, whereas kidneys from the remaining donor pool underwent nonoxygenated HMP. Belzer MPS solution (Bridge to Life, USA) was used as the perfusion solution for HMP, no other substances were supplemented. Donor kidneys that were assigned to the NMP intervention group were subjected to 2-hour NMP with a plasma-free red cell–based solution upon arrival at the Erasmus Medical Center. Needle kidney biopsies were collected before and after 2-hour NMP. The biopsies were snap-frozen in liquid nitrogen and stored at −80 °C. After 2-hour NMP, the kidneys were transplanted. This study was executed in compliance with the Declaration of Helsinki. All patients included in this research provided written informed consent, which was approved by the Medical Ethical Committee of Erasmus Medical Center (MEC 2020-0366).

### Single-Nuclei Isolation of Human Kidney Biopsies

Nuclei isolation and library construction were performed separately for each kidney biopsy. Kidney tissue was cut into small pieces on ice in a sterile petri dish using 1-ml nuclei isolation buffer (Nuclei EZ lysis buffer, N3408-200ML, Sigma-Aldrich) supplemented with Complete Protease Inhibitor Cocktail (1 tablet in 4 ml lysis buffer, 11836170001, Merck), 10 μl/ml RNase Inhibitor (Y9240L, Enzymatics) and 10 μl/ml Protector RNase Inhibitor (3335399001, Roche). Samples were homogenized using a dounce homogenizer (Tissue grind tube 885303-0002, pestle A 885301-0002, pestle B 885302-0002, Kimble), undergoing 10 to 15 strokes with each pestle. The lysates were then filtered through a 40 μm sieve and centrifuged at 4 °C, 500 g for 5 minutes. After centrifugation, the nuclei were resuspended in 200 μl sterile phosphate-buffered saline

(Bio&SELL) supplemented with 2% Ultrapure bovine serum albumin (AM2616, Thermo Fisher) and 2 μl/ml Protector RNase Inhibitor (3335399001, Roche). The nuclei were stained with 4′,6-diamidino-2-phenylindole (D9542-10MG, Sigma-Aldrich), and fluorescence-activated nuclei sorting was performed via Sony SH800 to enrich the nuclei.

### snRNA seq

For the snRNA seq, 18,000 to 20,000 nuclei per sample were loaded into the chromium controller (PN-120223, 10x Genomics) on a Chromium Next GEM Chip G (PN-2000177, 10× Genomics) and processed following the manufacturer’s original protocol. The library construction was performed using the Chromium Next GEM Single Cell 3ʹ GEM, Library and Gel Bead Kit v3.1 (PN-1000121, 10× Genomics) and Dual Index Kit TT Set A (PN-1000215, 10× Genomics). Quality control for the constructed library was performed using a High Sensitivity DNA Assay (5067-4626, Agilent technologies). Libraries were sequenced on NovaSeq6000 (Illumina) targeting 50,000 reads per cell.

### Preprocessing of snRNA seq

CellRanger (v6.0.2) was used to perform the alignment with standard parameters. The option “–include-introns” was activated because the input consisted of nuclei.

### Processing of snRNA seq Data

All data samples underwent initial checks for ambient RNA contamination using SoupX (v1.6.2).^15^ Genes labelled as ambient RNA were mitochondrial genes, which are unexpected in nuclei. Data analysis was processed with Seurat (v4.2.0),^16^ eliminating genes expressed in < 3 cells and cells with < 200 transcripts. Additional checks for doublets (with DoubletFinder^17^), normal relative gene expression across cells, and percentage of ribosomal genes were conducted.

RNA expression was normalized for sequencing depth per cell. Then data integration used reciprocal principal component analysis to address technical variations, followed by scaled to a uniform mean and SD and principal component analysis on the integrated data.

Cell clustering resolutions and the optimal clustering were manually selected with the aid of clustree (v0.5).^18^ Uniform manifold approximation and projection provided 2-dimensional data representation.

Lists containing differentially expressed genes within clusters were generated using presto (v1.0).^19^ Output genes were used for manual cluster annotation (Supplementary Table S1).^20^, ^21^, ^22^, ^23^, ^24^

Comparative analyses between groups (t0 vs. t2; DGF vs. non-DGF) were performed using cell composition differences and gene expression shifts.

For the compositional data analysis, the nonindependent cell proportions were compared between groups using methodology described previously.^25^

For the gene expression shift analysis, cell types with < 10 cells were excluded. Then, a “pseudo-bulk” matrix was generated based on the gene expression counts across all cells. Pairwise distances between genes were estimated. Subsequently the expression shift was estimated by normalizing the pairwise distances and adjusting them for the expected amount of change under permutation of sample groups. Using this approach, snRNA seq data were used to perform *in silico* normalization across cell populations, which discards gene expression variability of the cells within each sample.

For differential expression analysis, DESeq2 (1.38.3)^26^ was used. Wald test was used per cell type. Significant DE lists per cell type were then used for pathway enrichment using fgsea (v1.24)^27^ using default settings. The database of the canonical pathways from the C2 (curated gene sets) collection from GSEA was used. Dot plot visualization of the pathways shows significant pathways (adjusted *P* < 0.001) per cell type with their respective normalized enrichment score.

Gene dot-plots represent the percentage expression by dot diameter and average non-0 expression in log2 scale by dot color; data were not shown if the percentage of expression was < 0.01.

Ligand receptor analysis between groups and cell types was carried out using CellChat (v1.5).^28^ All cell-cell pairs that were present in < 10 cells were omitted.

### qPCR

RNA of kidney biopsies was isolated using RNeasy Mini Kit (Qiagen). The quantity of RNA was measured using DeNovix DS-11 (DeNovix). cDNA was synthesized from 200 ng RNA in 40 μl reaction volume through Moloney Murine Leukemia Virus Reverse Transcriptase Kit (M-MLV RT; Invitrogen), random primers (Promega), and RNasin ribonuclease inhibitor (Promega). qPCR was performed with TaqMan Gene Expression Master Mix (Thermo Fisher) and TaqMan Gene Expression Assays (Thermo Fisher) (Supplementary Table S2). Gene expressions were calculated using 2^−ΔΔCT^ method expressed as a fold change, using *GAPDH* as housekeeping gene. Significance levels were estimated by a permutation test (with BH adjustment) and are indicated by a star on the top.

Correlation between clinical variables and gene expressions was assessed, with normality confirmed using Shapiro-Wilk tests. Pearson analysis was applied to normally distributed variables, whereas Spearman analysis was used for others. A 2-tailed *P*-value < 0.05 indicated statistical significance.

## Results

### snRNA seq of Donor Kidney Biopsies Reveals all Major Kidney Cell Types

**Figure 1:**
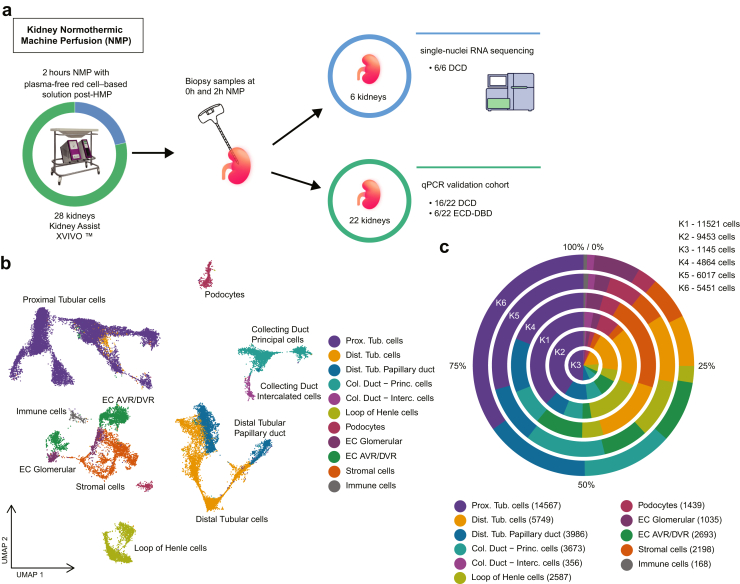
Single nucleus RNA sequencing of kidney biopsies taken before and 2 hours after NMP. (a) Schematic presentation of the experimental setup. Six DCD kidneys were subjected to 2-hour NMP and single nucleus RNA sequencing performed on biopsies taken at 0-hour and 2-hour after NMP. (b) Unsupervised clustering of cells of kidney biopsies collected after NMP (*n* = 12 biopsies from 6 kidneys, *n* = 38,451 cells) and identification of 11 renal cell clusters. (c) Distribution of the 11 renal cell clusters per sample; no major compositional differences were observed across samples. DCD, donation after circulatory death; DBD, donation after brain death; EC, endothelial cells; EC AVR/DVR, ECs of ascending vasa recta/descending vasa recta; ECD-DBD, extended criteria DBD; HMP, hypothermic machine perfusion; NMP, normothermic machine perfusion; qPCR, quantitative real-time polymerase chain reaction; UMAP, uniform manifold approximation and projection.

Biopsies of kidneys 1 to 6 were subjected to snRNA seq both before and upon completion of 2-hour NMP (Figure 1a). Three of the kidneys demonstrated DGF and 3 were non-DGF kidneys (Table 3). A total of 38,451 cells comprising 11 different cell types were identified after stringent quality control analysis and subsequent clustering (Figure 1b). Each of the 6 kidneys contributed to the diverse populations of cell types (Figure 1c). There were no differences in cell numbers of each cell type before and after NMP (Supplementary Figure S1).

### NMP Changes Gene Expression Profiles in Most Cell Types of the Kidney

**Figure 2:**
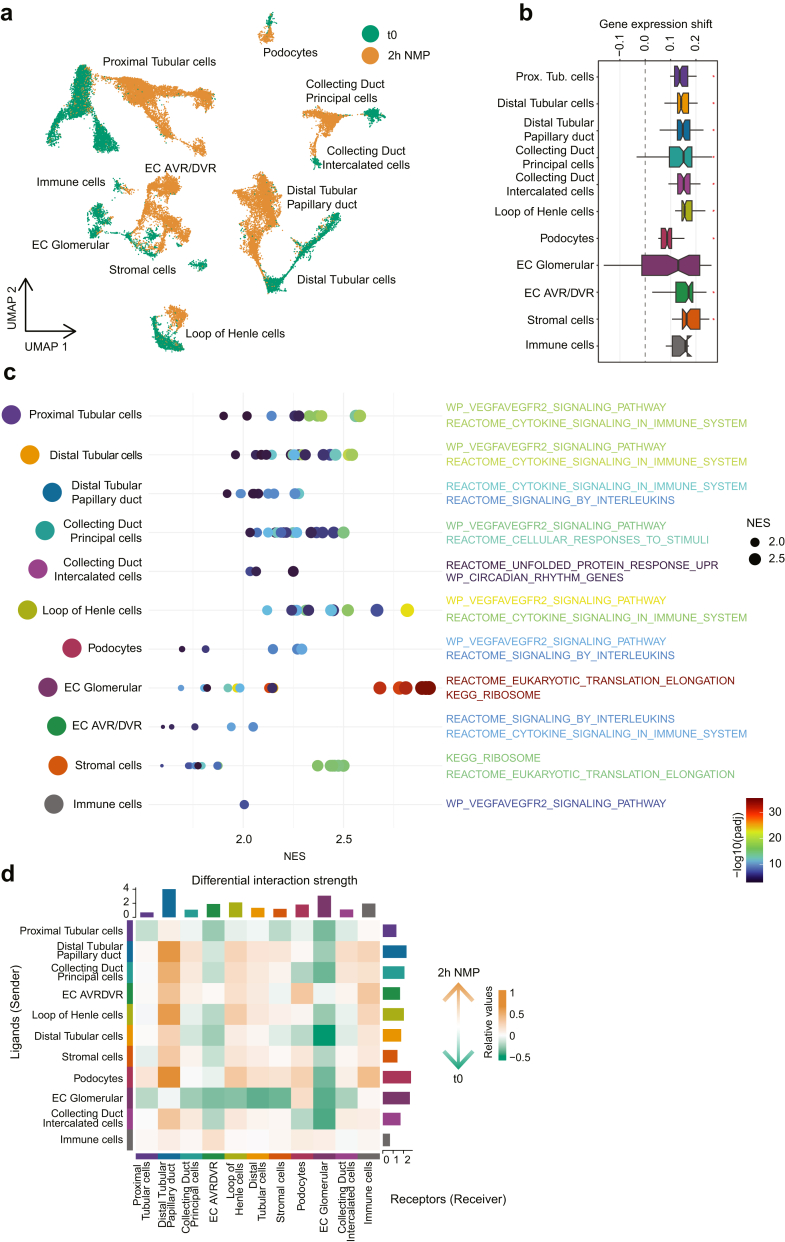
Effect of NMP on gene expression of kidney cell types. (a) Unsupervised clustering of 11 renal cell clusters showed a shift in position in the reduced dimensionality space after NMP. (b) Boxplot shows gene expression shifts between 0 and 2 hours after NMP (y-axis) for all cell types. Significance levels are estimated by a permutation test, and *P* < 0.05 after Benjamini-Hochberg correction is indicated by ∗. (c) Overview of significantly upregulated pathways in various cell types. Both the adjusted *P*-value and the NES are displayed. (d) Heatmap displays the differential interaction strength between cell types from the ligand-receptor analysis between 0 and 2 hours after NMP. The checkerboard represents the sum of column(ligand)/row(receptor) values displayed in the heatmap. EC, endothelial cells; EC AVR/DVR, ECs of ascending vasa recta/descending vasa recta; NES, normalized enrichment score; NMP, normothermic machine perfusion; UMAP, uniform manifold approximation and projection.

Next, differential gene expression profiling of various cell types before NMP and 2 hours after NMP was performed. Apart from glomerular endothelial cells (ECs) and immune cells, all cell types of the kidney responded to 2-hour NMP with gene expression changes (Figure 2a and b) (Supplementary Figure S2). In particular, genes of HSP family such as *HYOU1*, *DANJA1*, *HSPH1*, and *HSPA4L*; proinflammatory genes such as *STAT3*, *NFKB1*, and *TGFB1*; ubiquitin-related gene, *UBE2H*; and ATP-related genes, *GOT1* and *SLC9A1*, were significantly upregulated in the majority of the cell types (Supplementary Figure S3). Moreover, elevated expression of genes involved in other biological processes such as angiogenesis and activation of cytokines and interleukins after NMP were observed (Figure 2c). Although glomerular ECs showed no significant gene expression shifts, they exhibited significant upregulation of the protein synthesis pathway and related genes.

To reveal the effect of NMP on potential interactions between different cell types, an analysis based on receptor-ligand interactions using CellChat was performed. Compared with samples collected before NMP, increased interactions between distal tubular-papillary ducts and most other cell types were found after NMP. In addition, increased interactions between immune cells and the majority of other cellular types were observed. Conversely, cell cross-talk of ascending vasa recta or descending vasa recta ECs and glomerular ECs with other cell types was downregulated after NMP (Figure 2d and Supplementary Figure S4).

### Gene Expression Profiles After NMP are not Associated With DGF

**Figure 3:**
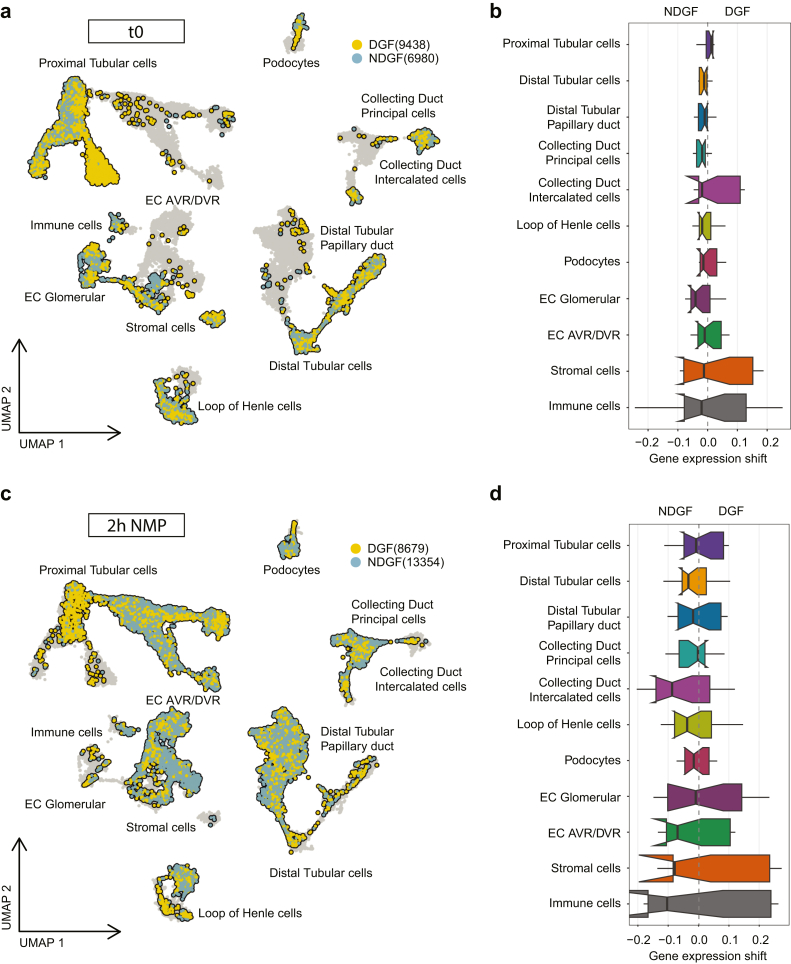
No difference in gene expression between DGF and NDGF kidneys before and after 2-hour NMP. (a) UMAP representation of cells from DGF and NDGF grafts before NMP. (b) Box plot represents distribution of normalized pairwise distances before NMP. (c) UMAP representation of cells from DGF and NDGF grafts after 2-hour NMP. (d) Box plot represents distribution of normalized pairwise distances after 2-hour NMP. DGF, delayed graft function; EC, endothelial cells; EC AVR/DVR, ECs of ascending vasa recta/descending vasa recta; NDGF, non-DGF; NMP, normothermic machine perfusion; UMAP, Uniform manifold approximation and projection.

We next analyzed whether the 3 kidneys with DGF displayed differential gene expression patterns compared with 3 non-DGF kidneys. Uniform manifold approximation and projection visualization showed that cell types from DGF or non-DGF samples did not cluster differently either before or after 2-hour NMP (Figure 3a and c). This was confirmed by the absence of gene expression shifts for the different cell types between DGF and non-DGF (Figure 3b and d).

### Gene Expression Profile After NMP Reflects Poor Posttransplant Outcome

**Figure 4:**
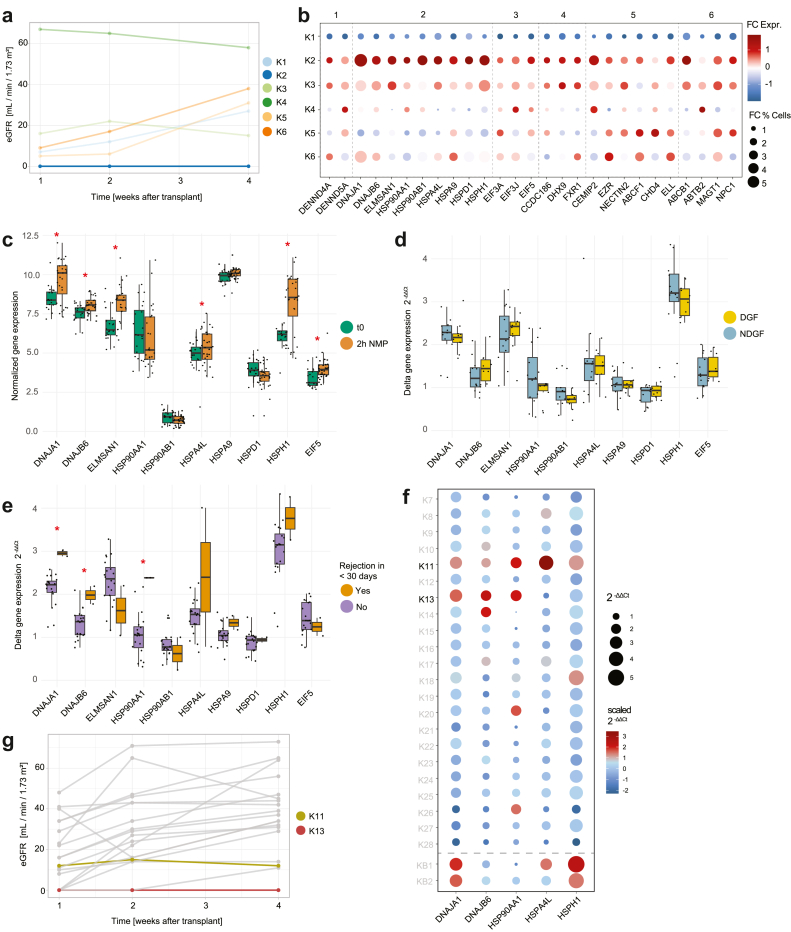
Deviated gene expression profiles after 2-hour NMP reveal poor posttransplant outcome. (a) eGFR measurements from the 6 kidneys analyzed by single nucleus RNA sequencing cohort in the first 4 weeks after transplantation. (b) FC of the most significantly different genes before and after NMP in snRNA seq cohort. Dot size denotes the percentage of cells expressing the genes. Square 1 shows the genes that encode guanine nucleotide exchange factors, square 2 shows genes that encode heat shock proteins, square 3 shows genes that encode ribosomal proteins and translation regulators, square 4 shows genes that encode RNA-related factors, square 5 shows genes that encode signaling and adhesion proteins, and square 6 shows transporter genes. (c) Normalized gene expression of most significantly different genes before and after 2-hour NMP in the qPCR cohort. The lower and upper edges of the box denote the first quartile (Q1) and third quartile (Q3), respectively. The median is marked with a line inside the box. The whiskers indicate 1.5 times the IQR. The notch indicates the confidence interval for the median's true value. (d) Normalized gene expression between DGF and NDGF kidneys under 2-hour NMP in the qPCR cohort. (e) Normalized gene expression between kidneys with rejection within 1 month and other kidneys under 2-hour NMP in the qPCR cohort. (f) Delta expression of most significantly different genes after 2-hour NMP in the qPCR cohort. (g) eGFR measurements from the cohort of 22 kidneys used for qPCR analyses in the first 4 weeks after transplantation; kidneys with low eGFR and rejection are highlighted. DGF, delayed graft function; eGFR, estimated glomerular filtration rate; FC, fold change; IQR, interquartile range; NDGF, non-DGF; NMP, normothermic machine perfusion; qPCR, quantitative real-time polymerase chain reaction.

To determine the functionality of kidneys 1 to 6 in the initial period after transplantation, eGFR was measured from day 0 to day 30 after transplantation at weekly intervals (Figure 4a). Kidney 2 showed no function and the recipient was on dialysis from days 0 to 30, and additionally experienced an acute rejection episode on day 8 (Table 3). We observed that for this specific kidney, there were several groups of genes that showed more pronounced upregulations during NMP in most cell types compared with other kidneys; especially, proteins in the HSP family were highly upregulated in kidney 2 (Figure 4b and Supplementary Figure S5).

### Validation of Findings by qPCR in 22 Kidneys

#### Validation of HSP Gene Expression in the qPCR Cohort

To investigate whether pronounced upregulation of HSP during NMP can be observed in other kidneys with poor posttransplant function, we measured the expression of a selection of the genes, mostly including HSP genes, using qPCR in biopsies from another 22 DCD or ECD-DBD kidneys after NMP. In concordance with the snRNA seq data, 6 out of 10 tested genes presented elevated expression during NMP in the qPCR cohort (Figure 4c). Similar to the snRNA seq data, no significant differences in the response to NMP were found between DGF and non-DGF kidneys (Figure 4d).

#### Subgroup Analysis for Male DCD Donor Kidneys

To assess the generalizability of the snRNA seq findings in male DCD donors, we performed a subgroup analysis of the qPCR cohort, focusing exclusively on male DCD donor kidneys, which mirrored the characteristics of the snRNA seq cohort. Gene expression profiles after 2 hours of NMP were not different in male DCD donor kidneys compared with the whole cohort (Supplementary Figure S6).

#### Urine Output Differences During NMP in DGF Versus Non-DGF Kidneys

We compared urine output at 60 and 120 minutes during NMP perfusion between DGF and non-DGF together with snRNA seq cohort. Urine output was significantly lower in DGF kidneys at both 60 minutes and 120 minutes than in non-DGF kidneys (Supplementary Figure S7). It suggests that reduced urine production during NMP may reflect impaired tubular function.

#### HSP Expressions in Kidneys With Early Rejection

Comparison of kidneys that experienced an episode of acute rejection in the first 30 days after transplantation with kidneys that were not rejected in this period demonstrated a significant upregulation of 3 HSP genes (*DNAJA1*, *DNAJB6*, and *HSP90AA1*) (Figure 4e). The 2 kidneys that experienced rejection in the first 30 days (kidneys 11 and 13) displayed an exaggerated response to NMP for a selection of 5 HSP-related genes that was similar to the response observed for kidney 2 with snRNA seq. HSP gene expression analysis was performed on 2 kidneys that underwent NMP but were discarded for transplantation because of prolonged ischemia and acute kidney injury. In these kidneys, HSP genes, were significantly elevated as well (Figure 4f).

Kidneys 11 and 13 showed a poorer kidney function after transplantation compared with other kidneys, evidenced by lower eGFR up to day 30 (Figure 4g), albeit there were other kidneys that showed rejection (kidneys 15, 18, and 27). Kidneys 11 and 13, however, were the only kidneys of this cohort that experienced an episode of acute rejection during the first 10 days after transplantation.

To better characterize the dynamic trajectory of graft function beyond static eGFR measurements, we performed a slope analysis of eGFR changes over time. As shown in Supplementary Table S3, most kidneys with favorable outcomes exhibited positive eGFR slopes, indicating progressive functional improvement. In contrast, kidneys 2, 11, and 13 showed minimal or flat trajectories (slope = 0, 0.32, and 0, respectively), indicating little to no recovery. Several other kidneys (kidney 21, 23, and 25) also had flat slopes, consistent with persistent dysfunction or failure.

#### Association Between Donor or Transplant Characteristics and HSP Gene Expression

Finally, to address whether donor and transplant characteristics affect transplant kidney gene expression, correlation analysis was performed. Donor hypertension was significantly correlated with downregulated *HSP90AB1* and *HSPD1* and upregulated *HSP90AA1*. Donor creatinine was negatively correlated with *HSP90AB1*, and urine production was negatively correlated with *ELMSAN1* (Supplementary Table S4).

## Discussion

Our study demonstrates that the major kidney cell types such as tubular cells, podocytes, and ECs show a robust change in gene expression profiles in response to NMP. Interestingly, 3 kidneys with poor posttransplant function displayed a differential HSP gene expression profile during NMP.

snRNA seq analyses demonstrated a large number of differentially expressed genes in kidneys after 2 hours of NMP. Important gene transcript signatures included the increased expression of genes related to ATP production, suggesting that NMP allows the kidney to replenish ATP and support metabolism. Such activation of the kidney during NMP is supported by earlier studies, including our finding that the endocrine system of the kidney is activated during NMP.^9^^,^^29^ Therefore, there is strong evidence that NMP facilitates physiological activity of the kidney. Additional pathways impacted by NMP included angiogenesis, which might indicate that reparative processes are activated in the kidney during NMP.

However, genes associated with inflammation were found to be elevated in response to NMP. This is likely an expression of ischemia reperfusion injury initiated during NMP. This is confirmed in other studies that inflammatory factors such as interleukin-1β, interleukin-6, and interleukin-8 were significantly increased during NMP.^30^^,^^31^ Therefore, removing donor-derived inflammatory mediators during NMP may help to reduce the inflammatory response after transplantation. Indeed, studies in both human and porcine kidney NMP showed that cytosorb adsorber can reduce the inflammatory response and improve renal blood flow during perfusion.^12^^,^^32^

We observed shifts in kidney cellular interactions during NMP. In particular, the interactions between distal tubular-papillary ducts and immune cells with other cell types were increased after NMP, suggesting that these cell types were activated by NMP. The potent effect of NMP on distal tubular-papillary ducts is a surprising finding because these cells are not known to represent the most responsive part of the tubule to ischemic and reoxygenation challenges. The upregulated receptor-ligand interactions of immune cells with other cells indicate an increase in chemotactic mobility of immune cells within the kidney and an activation of other cell types by inflammatory signals derived from immune cells. This may contribute to an increased inflammatory state of the kidney after NMP. In contrast, ECs, both glomerular and ascending vasa recta or descending vasa recta–derived, showed reduced interactions with other cell types. It is not yet clear whether this observation points to quiescence of these cells or whether it reflects cellular injury during NMP.

An interesting observation of our study was the increased expression of a variety of HSP and related genes during NMP. HSPs play a protective role in cellular stress responses.^33^ They assist in facilitating protein formation and participate in the processes of repair or removal of damaged or denatured proteins or their toxic aggregates.^34^ Therefore, our results show that a 2-hour NMP not only triggers stress responses, but also activates protective pathways that enhance cell survival and proliferation. HSPs that are produced during NMP may help to condition the kidney in preparation for reperfusion. This suggests that a short period of NMP after cold ischemia may promote recovery immediately after transplantation.^35^

Although activation of cell-protective genes during NMP may be beneficial, our results demonstrate that an overabundance of HSP family genes is associated with poor posttransplant outcome. We found that during NMP, HSP genes were significantly elevated in 3 kidneys that showed acute rejection within the first 10 days after transplantation. Of these kidneys, 2 remained nonfunctional, whereas the recipient of the third kidney died on day 33 due to urosepsis as a result of pyelonephritis with abscess. Moreover, the elevated HSP gene expression in 2 kidneys that were discarded because of long ischemia times and acute kidney injuries suggests that exaggerated HSP expression during NMP may be related to poor kidney outcome. Furthermore, it potentially provides information that can be used to determine whether a kidney should be accepted for transplantation. Another piece of evidence is that serum HSP90α level has been shown to significantly increase in kidney recipients at the time of acute rejection compared with those with no evidence of rejection.^36^

In our cohort, there were 3 other kidneys (kidneys 15, 18, and 27) with an episode of acute rejection after transplantation that showed similar HSP expression after NMP as kidneys without rejection. Interestingly, rejection in these kidneys occurred at least 90 days after transplantation, suggesting that the HSP profile after NMP is deviant only in kidneys with early rejection but is not discriminative for kidneys with late rejections. At later time points, posttransplant factors such as infections, recurrence of disease, immunosuppression, and adherence play increasingly important roles. Although kidney 6 also experienced early rejection, it demonstrated a gradual recovery of eGFR. Our data suggest that gene expression profiles during NMP provide a signature for kidneys that reject early after transplantation and have a poor recovery of kidney function.

In addition, in our study, donor clinical outcomes such as hypertension and creatinine correlated with the expression of 3 HSP genes. These genes are thus not only involved in cellular stress responses, but they are also related to the initial status of the donor kidneys. Although we evaluated the discriminatory ability of individual genes in identifying early rejection together with poor functioning cases, future studies could focus on increasing the number of samples, so as to further confirm the cut-off value of multiple HSP profiles in predicting allograft loss, thus providing a basis for the development of rapid diagnostics such as test strips, enzyme-linked immunosorbent assay, and fast PCR. Overall, nontransplantable kidneys may be well-differentiated by combined HSP analysis and other measurements, thus preventing patients from experiencing early graft failure, which may lead to secondary transplantation, and avoiding unnecessary discard of kidneys that may function normally after transplantation.

It is known that elderly donors (aged > 60 years) are at an increased risk of DGF.^37^ Consistently, in our study, 13 of the included patients presented with DGF, 9 of whom were aged > 60 years. No significant differences in gene expression related to DGF were noted before and after NMP, suggesting that DGF may not be strongly associated with transcriptional stress responses detectable during a 2-hour NMP session. Alternatively, NMP duration may be insufficient to reverse cold ischemic injury at the transcriptional level, even if it improves ATP levels and homeostasis. However, our findings are different from a recent study whose results showed a significantly lower DGF incidence in kidneys receiving NMP compared with kidneys receiving only cold storage.^38^ This may be related to the lower age of the donors they included (median: 62 years vs. 49 years) and the shorter total ischemic time.

Finally, in our research, we opted for a 2-hour NMP protocol because a single-center pilot study verified both the feasibility and safety of a 2-hour NMP implementation.^39^ Although 1-hour NMP has been shown to induce multiple gene expression changes, shorter period of NMP may not be enough to differentiate good and poor-quality kidneys. Alternatively, longer periods of NMP may improve the discriminative potential between good and poor kidneys.^40^

This study had a number of limitations. First, the sample size for the snRNA seq and qPCR cohorts was limited. In particular, numbers of primary nonfunctional kidneys and kidneys with rejection were small. Second, because all donors were DCD or ECD-DBD, the study lacked kidneys of optimal quality for comparison. Despite limitations in sample size and a lack in diversity in donor kidney quality, our study offers credible findings on novel gene expression markers that provide insights beyond traditional perfusate analysis into the molecular mechanisms that capture organ-intrinsic quality and short-term resilience.

In conclusion, our study demonstrates that 2-hour NMP impacts gene expression profiles of most cell types of the kidney. Moreover, the cellular response to NMP is associated with posttransplant kidney function, providing new ideas for early detecting graft status.

## Disclosure

All the authors declared no competing interests.

### Funding Statement

The authors received grants from The 10.13039/501100002997Dutch Kidney Foundation (grant no. 19OI09 ‘Laser speckle imaging: a real-time tool for viability assessment of extended criteria donor kidneys during normothermic machine perfusion’) and 10.13039/501100004948Astellas Pharma. The findings and conclusions in this report are those of the authors and do not necessarily represent the official position of the funding agency.

## Data Availability Statement

All snRNA seq data generated in this study have been deposited in the NCBI’s Gene Expression Omnibus database (GEO GSE307631). qPCR data may be available from the corresponding author upon reasonable request.

## Author Contributions

SL and HT-M contributed to writing of original draft, review and editing, visualization, project administration, methodology, investigation, formal analysis, and conceptualization. JSS contributed to writing (review and editing), resources, and methodology. DMH-P, IC, YF, and SB contributed to resources and methodology. DS, AB, FS, and TA contributed to formal analysis and methodology. MEJR and RK contributed to writing (review and editing) and supervision. RCM contributed to funding acquisition, resources, methodology, conceptualization, project administration, supervision, visualization, and investigation. MJH contributed to writing (review and editing), conceptualization, supervision, project administration, methodology, investigation, and visualization.

## Supplementary Material

Supplementary File (PDF) **Figure S1.** Number of cells per cell cluster remains unchanged after NMP. **Figure S2.** Differential expressed genes per cell type due to NMP. **Figure S3.** Expression of inflammatory/ ATP related/ heat shock protein/ ubiquitin gene expressions is higher during NMP. **Figure S4.** Signaling pathways with higher probability per cell type. **Figure S5.** Differential expressed genes per cell type due to NMP in kidney 2. **Figure S6.** Gene expression comparison between snRNA seq and qPCR cohorts in male DCD kidneys. **Figure S7.** Urine output during NMP in DGF versus non-DGF kidneys. **Table S1.** Genes used for cell cluster labelling. **Table S2.** Gene expression primers for qPCR. **Table S3.** Slopes of eGFR recovery across the early posttransplant period. **Table S4.** Correlation analyses between gene expression changes induced by 2-hour NMP and clinical outcomes.
