# Supplementary material for: Cellular Responses During Kidney Normothermic Machine Perfusion Reflect Graft Outcomes
Source: Kidney Int Rep. 2025 Aug 20;10(11):4012–26. doi: 10.1016/j.ekir.2025.08.017 (PMC12639818; doi:10.1016/j.ekir.2025.08.017)
Supplement: Supplementary File (PDF) — Figure S1. Number of cells per cell cluster remains unchanged after NMP. Figure S2. Differential expressed genes per cell type due to NMP. Figure S3. Expression of inflammatory/ ATP related/ heat shock protein/ ubiquitin gene expressions is higher during NMP. Figure S4. Signaling pathways with higher probability per cell type. Figure S5. Differential expressed genes per cell type due to NMP in kidney 2. Figure S6. Gene expression comparison between snRNA seq and qPCR cohorts in male DCD kidneys. Figure S7. Urine output during NMP in DGF versus non-DGF kidneys. Table S1. Genes used for cell cluster labelling. Table S2. Gene expression primers for qPCR. Table S3. Slopes of eGFR recovery across the early posttransplant period. Table S4. Correlation analyses between gene expression changes induced by 2-hour NMP and clinical outcomes. [file mmc1.pdf]

Supplementary Materials

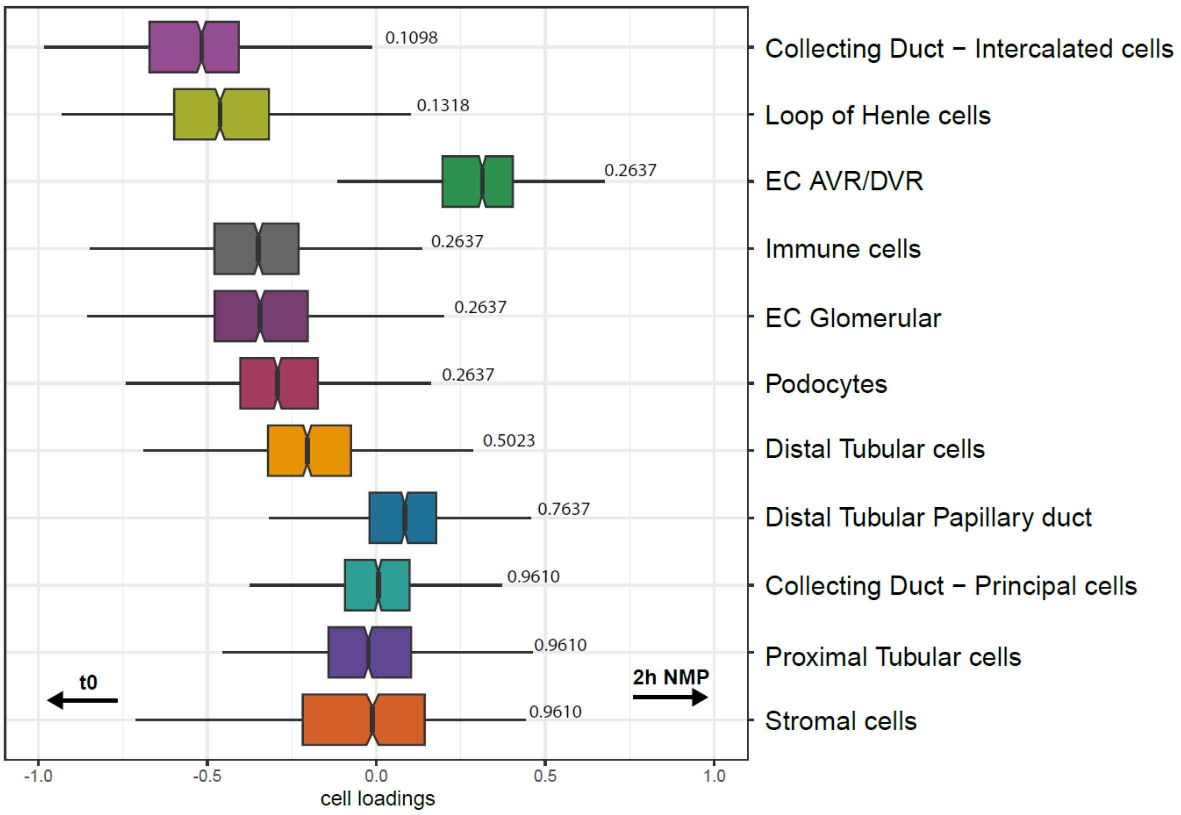

Supplementary Figure S1. Number of cells per cell cluster remains unchanged after NMP.

Positive loadings correspond to over-representation at 2 h NMP and negative loadings mean over-representation at 0 h NMP. The boxplots show how much the cell loading coefficients vary in the dataset. P value is indicated next to the boxplot.

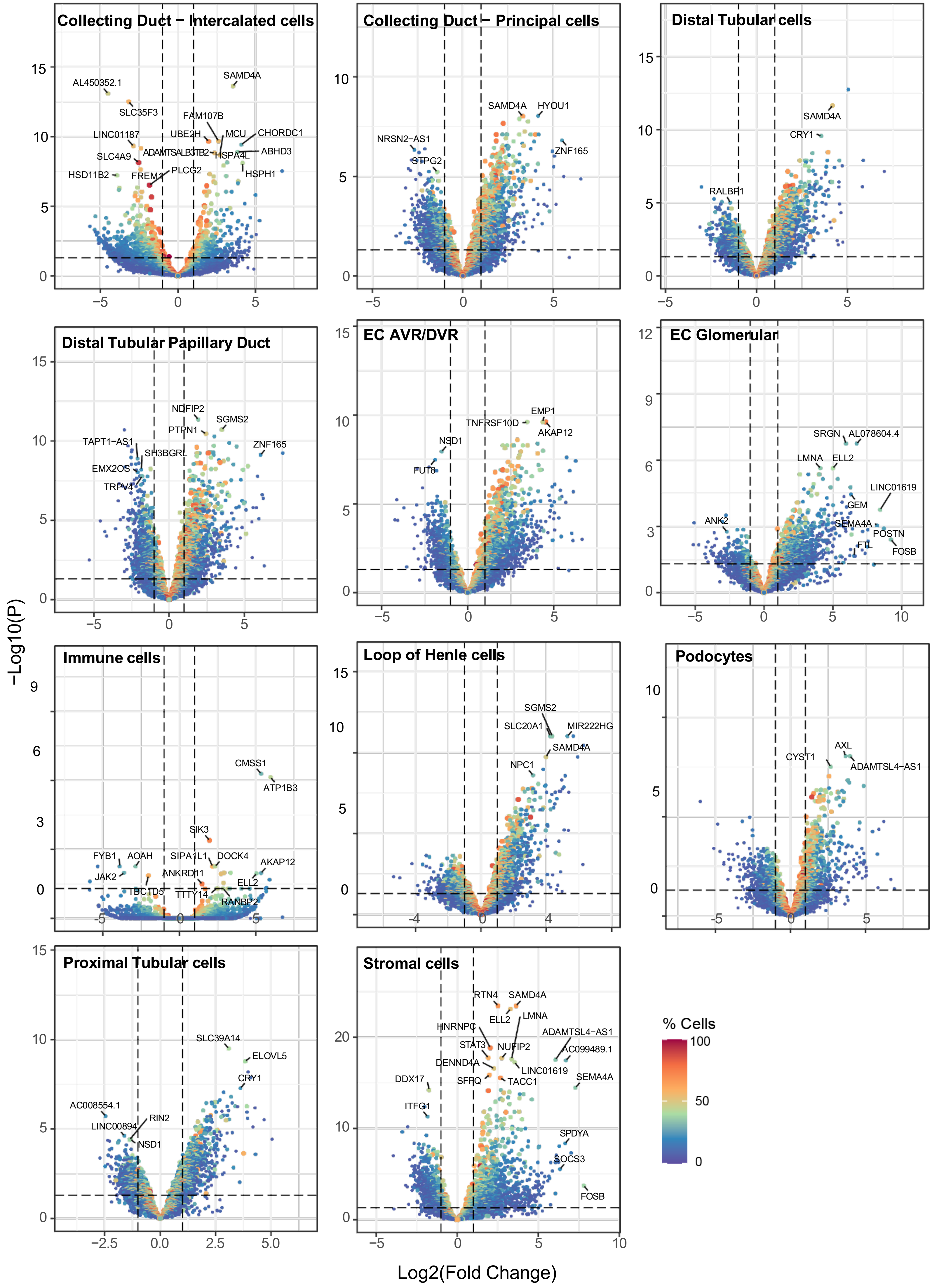
Collecting Duct − Intercalated cells
AL450352.1
SAMD4A
SLC35F3
FAM107B
LINC01187
UBE2H
MCU
CHORDC1
ADAMTSL4
ABHD3
SLC4A9
HSPA4L
PLCG2
HSD11B2
FREM1
HSPH1
Collecting Duct − Principal cells
SAMD4A
HYOU1
NRSN2−AS1
ZNF165
STPG2
Distal Tubular cells
SAMD4A
CRY1
RALBP1
Distal Tubular Papillary Duct
NDFIP2
SGMS2
PTPN1
TAPT1−AS1
ZNF165
SH3BGRL
EMX2OS
TRPV4
EC AVR/DVR
EMP1
TNFRSF10D
AKAP12
NSD1
FUT8
EC Glomerular
SRGN
AL078604.4
LMNA
ELL2
LINC01619
GEM
ANK2
SEMA4A
POSTN
FTL
FOSB
Immune cells
CMSS1
ATP1B3
SIK3
FYB1
AOAH
SIPA1L1
DOCK4
AKAP12
JAK2
ANKRD11
ELL2
TBC1D5
TTTY14
RANBP2
Loop of Henle cells
SGMS2
SLC20A1
MIR222HG
SAMD4A
NPC1
Podocytes
AXL
CYST1
ADAMTSL4−AS1
Proximal Tubular cells
SLC39A14
ELOVL5
CRY1
AC008554.1
RIN2
LINC00894
NSD1
Stromal cells
RTN4
SAMD4A
ELL2
LMNA
HNRNPC
ADAMTSL4−AS1
STAT3
NUFIP2
AC099489.1
DENND4A
LINC01619
DDX17
SFPQ
TACC1
SEMA4A
ITFG1
SPDYA
SOCS3
FOSB
−Log10(P)
Log2(Fold Change)
% Cells
100
50
0

Supplementary Figure S2. Differential expressed genes per cell type due to NMP.
Volcano plots showing changes in gene expression. The horizontal axis represents the logarithmic fold change in gene expression (log2(fold change)) and the vertical axis represents the negative logarithm of the p-value (-log(P)). Each dot represents a gene. Color scale represents the cell fraction. Thresholds are drawn at FC=1 and p-value = 0.05.

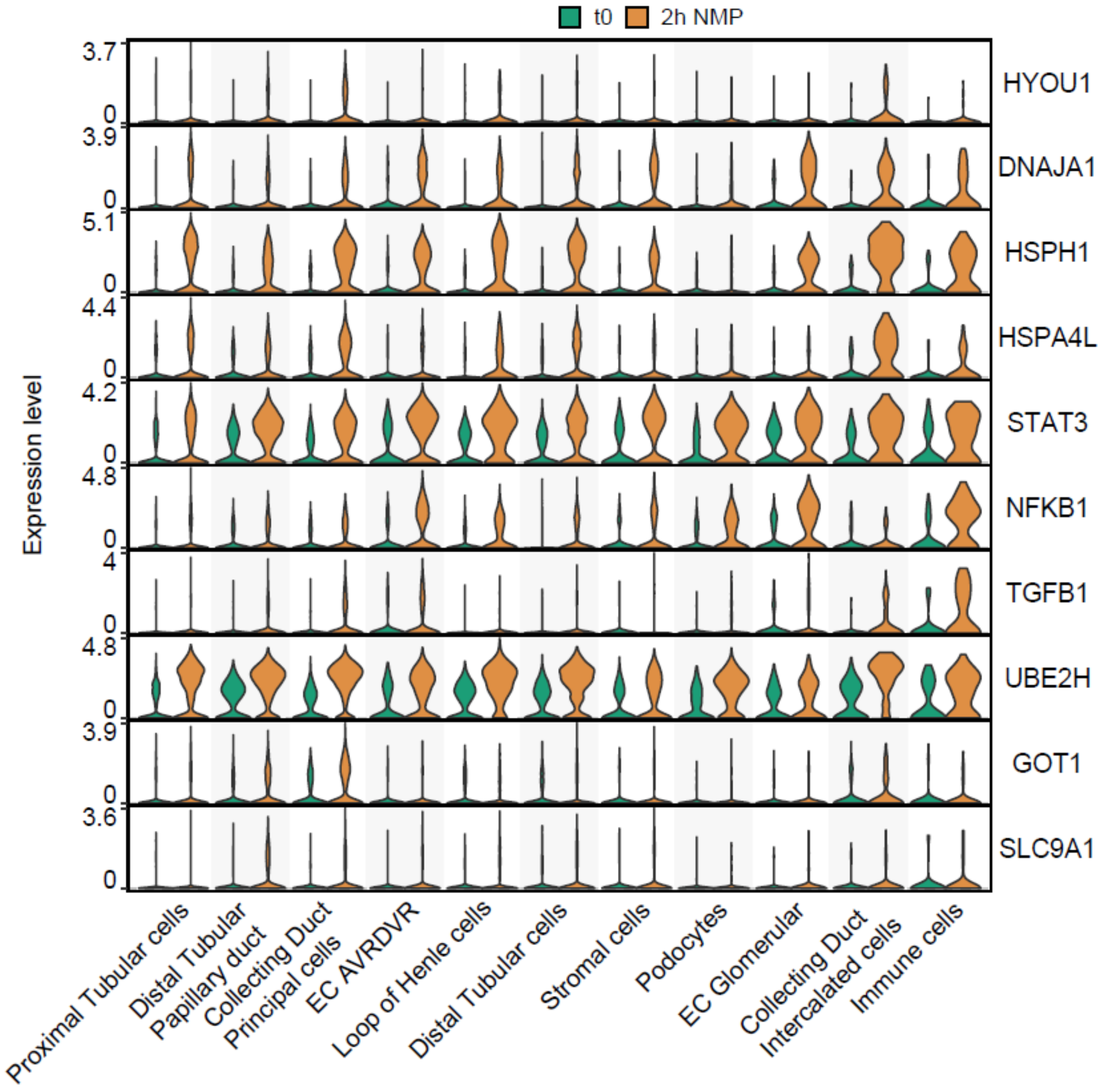

Supplementary Figure S3. Expression of inflammatory/ ATP related/ heat shock protein/ ubiquitin gene expressions is higher during NMP. Violin plots of selected genes in each cell cluster. The vertical axis shows the normalized gene expression level for each gene. 0 h NMP data are shown in green shades, while 2 h NMP data are shown in orange shades.

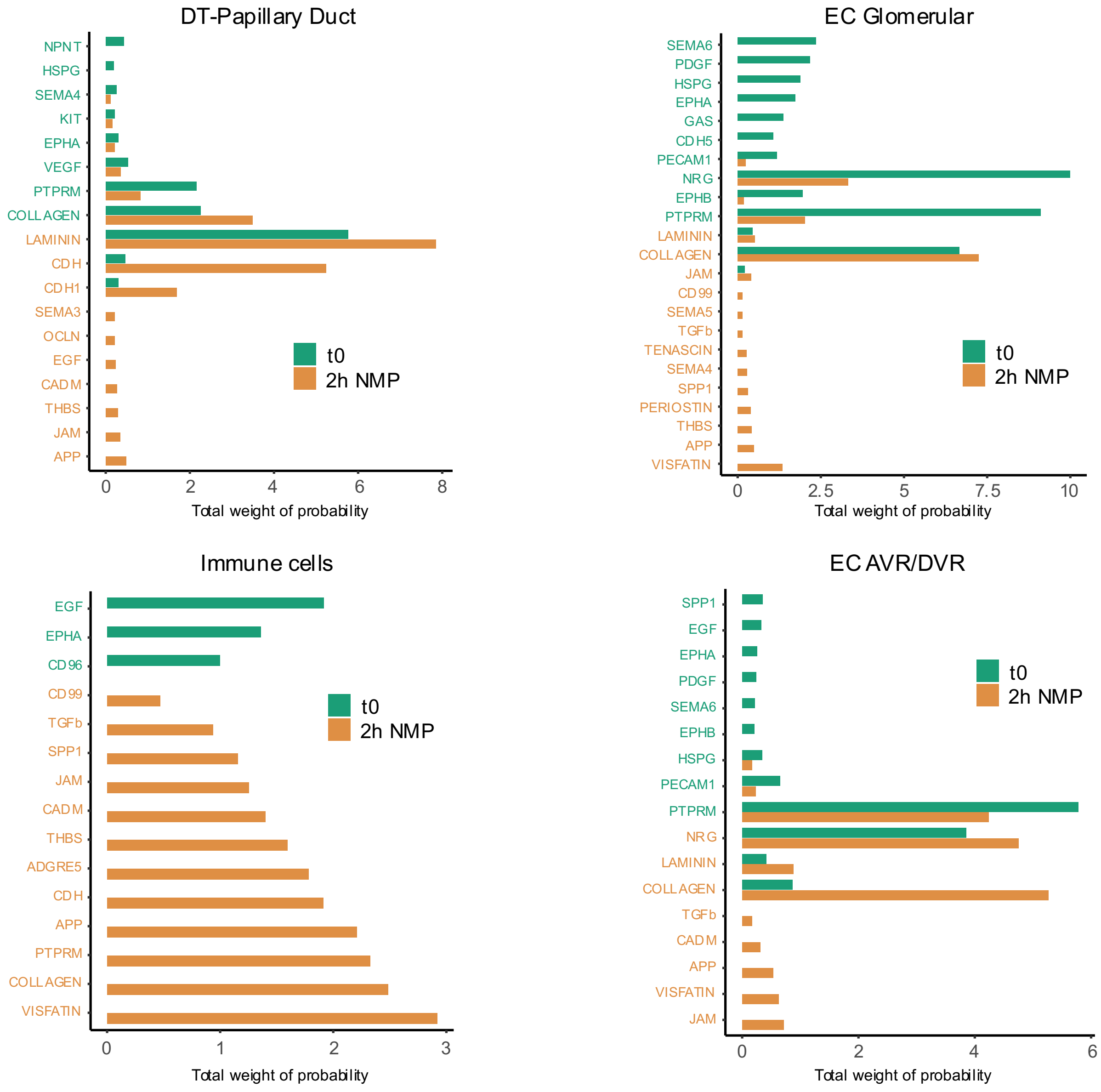

Supplementary Figure S4. Signaling pathways with higher probability per cell type. Bar charts of significant pathways ranked based on differences between t0 and 2h NMP. The total weight probability is defined as the sum of cell-cell interaction probability among all pairs in every cell type from the ligand-receptor network.

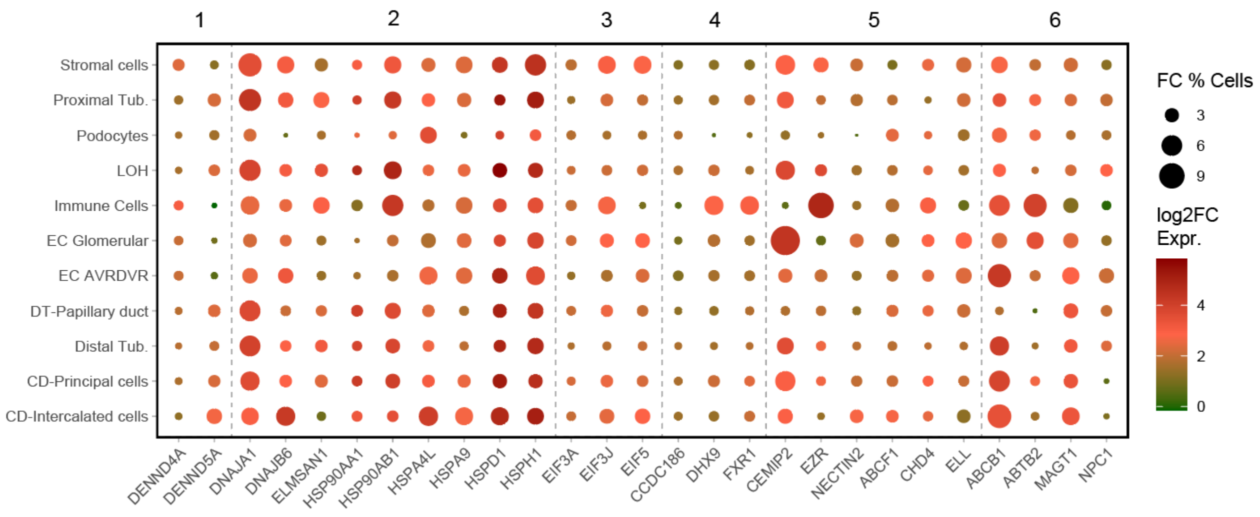

Gene Group

1. Guanine Nucleotide Exchange Factors
2. HSP genes & Chaperones
3. Ribosomal Proteins & Translation Regulators
4. RNA Related Factors
5. Signaling and Adhesion Proteins
6. Transporters

Supplementary Figure S5. Differential expressed genes per cell type due to NMP in Kidney 2.

Dotplot depicting the fold expression of significantly different genes before and after NMP in Kidney 2 sample. Dot size denotes the percentage of cells expressing the genes.

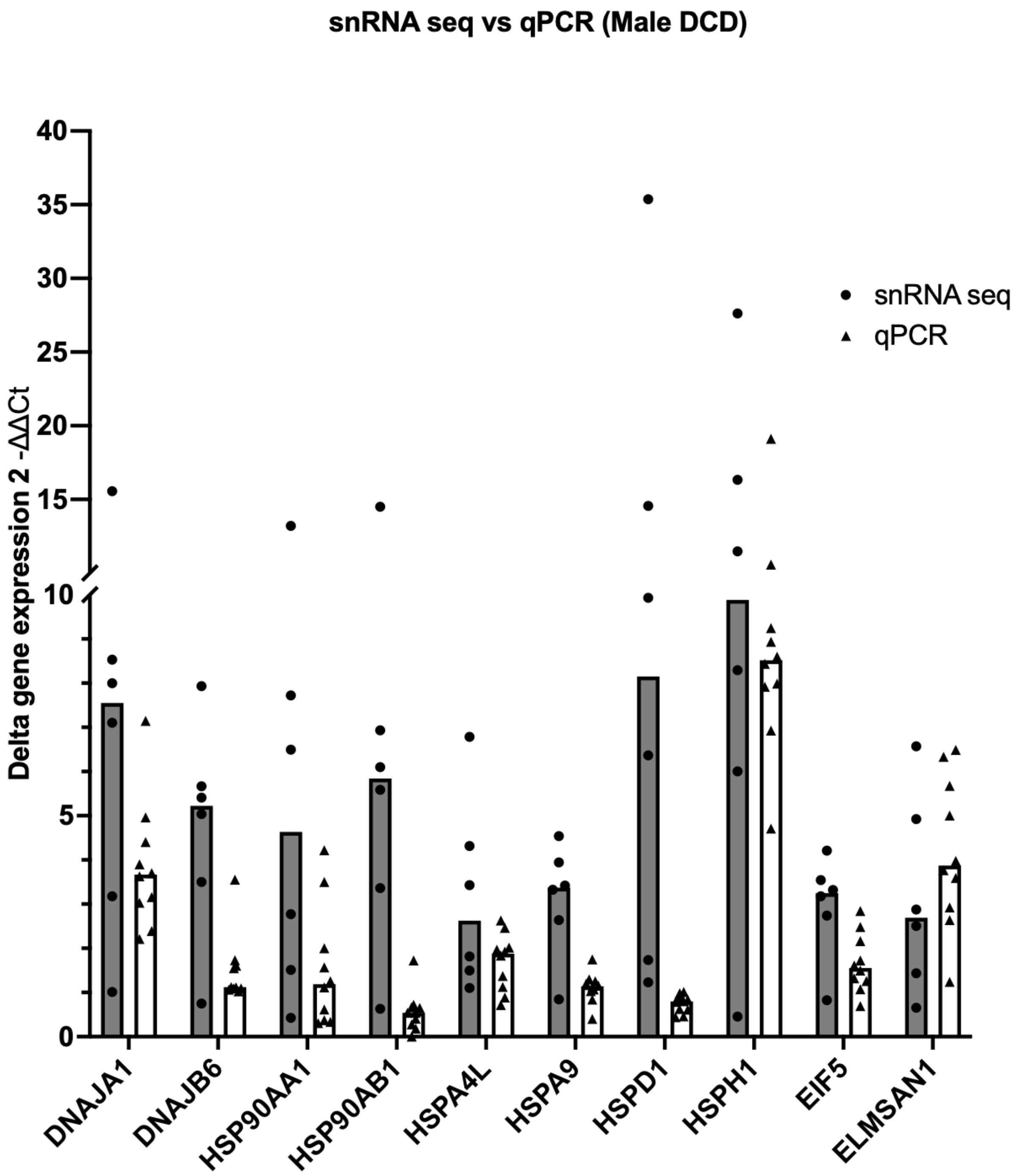

Supplementary Figure S6. Gene expression comparison between snRNA seq and qPCR cohorts in male DCD kidneys.

Data are represented as median with interquartile range. Each symbol represents a data point from 1 kidney sample. N=6 for snRNA seq cohort, 10 for qPCR cohort, 1 for early rejection group.

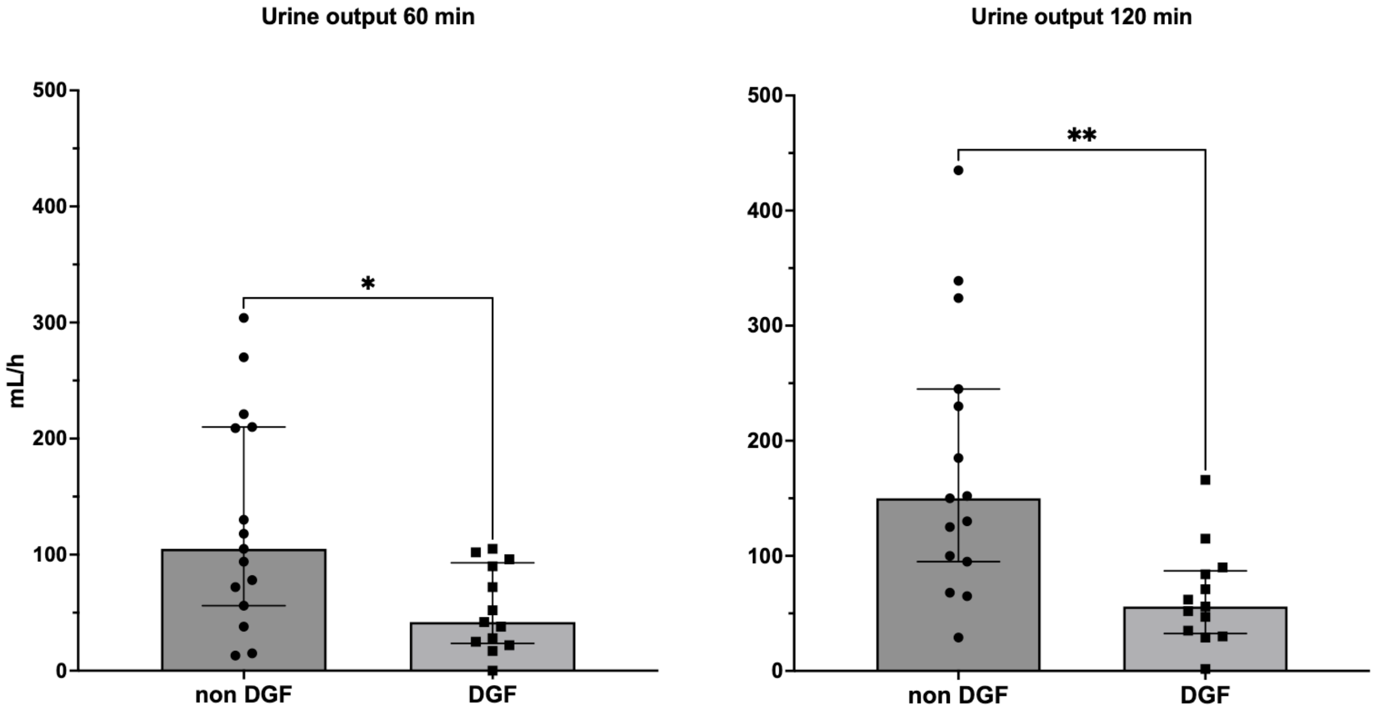

Supplementary Figure S7. Urine output during NMP in DGF vs. non-DGF kidneys. Data are represented as median with interquartile range. Each symbol represents a data point from 1 kidney sample. N=15 for non DGF group, 13 for DGF group. Asterisks reflect statistically significant differences (*P* < *0.05*).

CD-Intercalated cells	CD-Principal cells	Distal Tub.	DT-Papillary duct	EC AVR/DVR	EC Glomerular	Immune Cells	LOH	Podocytes	Proximal Tub.	Stromal cells
MAGI3	PTGER3	CLDN4	KAZN	AC119674.1	PLPP3	PTPRC	ESRRG	ST6GALNAC3	SLC27A2	IGFBP5
SLC35F3	RHBG	ATP1B1	COBLL1	PLPP3	EPAS1	ARHGAP15	CCDC148	NTNG1	SPP1	PTMA
TFCP2L1	ATP6V1C2	KRT19	AC019197.1	ST6GALNAC3	RAPGEF4	ANKRD44	PKP4	NFASC	SLC22A8	RBMS3
AC011246.1	MAGI1	GATA3	GLS	EPAS1	BMPR2	DOCK10	PDE1A	FMN2	PDZK1	ELL2
MAGI1	CLNK	ESRRG	MECOM	SH3RF3	IGFBP5	INPP5D	ERBB4	PLA2R1	LRP2	EBF1
CLNK	SLIT2	CACNB4	KCNIP4	BMPR2	RBMS3	MBNL1	CACNA2D3	PARD3B	PTH2R	SLIT3
PDZD2	ADGRF5	AC019197.1	ITGA2	LDB2	TGFBR2	DOCK2	CASR	COL4A3	SLC4A4	EEF1A1
SLC4A9	PACRG	SCN2A	ARL15	ARHGAP26	LDB2	CCND3	CCSER1	ABLIM2	ANK2	AKAP12
PACRG	PDE1C	ERBB4	PDE4D	AKAP12	EMCN	ELMO1	NPSR1-AS1	CLIC5	SLC17A1	ACTB
PDE1C	GALNT17	CACNA2D3	DCDC2	DOCK4	PLPP1	DOCK8	HIP1	DST	TINAG	CALD1
SLC26A4	ATP6V0D2	KCNIP4	CD2AP	TACC1	EBF1	FNBP1	LINC01606	MAGI2	CUBN	TACC1
DGKI	EGF	CCSER1	CADPS2	PLAT	SYNE1	CELF2	ENOX1	DPP6	CACNA1C	PRKG1
SLC26A7	KLHL3	ABTB2	GNA14	DOCK4	CAMK1D	GPC5	NEBL	ABCC2	A2M	CACNA1C
SHOC1	CACNB2	MALAT1	JAM3	PLAT	CHST11	PRKD1	PLCE1	SLC22A6	PLEKHA5	A2M
CELF2	PTPRJ	TRPM6	GRIP1	FLT1	ITGA8	PRKCB	SLC12A1	PTPRO	PAH	SLC38A2
NEBL	IL18	MALAT1	CDH1	PITPNC1	ZEB1	CYTH1	UMOD	PTPRQ	SYNE2	MEIS2
IL18	TMEM117	WNK1	SPIRE1	PTPRM	NRG3	SMCHD1	PLCB1	FRY	RAB11FIP3	B2M
CA12	NRXN3	TMEM52B	APCDD1L	TCF4	PTPRB	MYO1F	SIM2	NPAS3	ACSM2A	CRISPLD2
PLCG2	CA12	FMN1	APCDD1L-DT	ERG	FLT1	ADGRE5	ARHGAP6	ATP10A	ACSM2B	FOSB
PPM1E	CDH1	TRPM7	MID1	SHANK3	MEIS2	MYO9B	CLCN5	NPHS1	ACSF2	TIMP3
CDH1	SLC4A1	ADAMTS17	-	PECAM1	PECAM1	DOCK2	SLC9A3	PODXL	SLC13A3	COL1A1
TMEM213	AQP2	SLC12A3	-	ENG	CD34	CD4	POU3F3	VEGFA	FTCD	TIMP1
-	SCNN1A	LHX1	-	CDH5	ENG	ITGAX	IRX1	WT1	-	PDGFRB
-	AVPR2x	FGF13	-	-	KDR	ITGAM	-	PTPRO	-	VIM
-	-	-	-	-	-	CD86	-	-	-	-

Supplementary Table S1. Genes used for cell cluster labelling. Gene function of used key genes continues on following page.

Gene function of used key genes [18-22]:

- CD-Intercalated cells. SLC26A4 and SLC26A7: These genes are strongly associated with the intercalated cells of the collecting duct, which are responsible for acid-base balance in the kidney. CA12: Carbonic anhydrase XII is another marker frequently expressed in intercalated cells. CDH1 (E-cadherin): This gene is commonly expressed in epithelial cells, which include intercalated cells of the kidney. TFCP2L1: This transcription factor has been linked to the regulation of genes in kidney epithelial cells. MAGI1 and MAGI3: These genes are associated with tight junctions and are expressed in epithelial cells, specially intercalated cells.
- CD-Principal cells. AQP2 (Aquaporin 2) and AVPR2 (Vasopressin Receptor 2) are classic markers of Principal Cells in the Collecting Duct. These cells are responsible for water reabsorption and are regulated by vasopressin. SCNN1A encodes the alpha subunit of the epithelial sodium channel (ENaC), which is also characteristic of Principal Cells. CA12 (Carbonic anhydrase XII), SLC4A1 (solute carrier family 4, member 1), are often associated with the epithelial characteristics of these cells. KLHL3 is involved in ion transport regulation, which is crucial for the function of the Principal Cells in controlling sodium and water balance.
- Distal Tub. SLC12A3: This gene is a well-known marker of distal tubule cells, involved in sodium and chloride transport. TRPM6 and TRPM7: These genes are associated with magnesium transport, which is a key function of the DT cells. WNK1: This gene is involved in the regulation of ion transporters, particularly in the DT cells. CACNA2D3, CACNB4, SCN2A: These are related to calcium and sodium channels, relevant to DT cells' function.
- DT Papillary Duct. COBLL1: Involved in actin cytoskeleton organization, which is crucial for the structural integrity of epithelial cells in the collecting ducts. GLS: Involved in glutamine metabolism, which is important for energy production in distal tubular cells. ITGA2: Encodes an integrin important for cell adhesion, which is critical in maintaining the structure of epithelial cells lining the collecting duct. JAM3: Involved in maintaining the integrity of tight junctions, which are important in the epithelial cells of the collecting ducts for controlling paracellular transport.
- EC AVR/DVR. PECAM1 (CD31), CDH5 (VE-cadherin), ENG (Endoglin), and FLT1 (VEGFR1) are classic markers of endothelial cells. BMPR2 is associated with endothelial cells and their response to BMP signaling. EPAS1 (HIF2A): This gene is important in hypoxia response and is particularly relevant to the kidney medulla, where oxygen tension is low. DVR endothelial cells, which operate in a hypoxic environment, would likely show upregulation of this gene. ERG: This is also typically expressed in endothelial cells involved in vascular functions.
- EC Glomerular. Key genes such as PECAM1 (CD31), CD34, KDR (VEGFR2), FLT1 (VEGFR1), and ENG (Endoglin) are well-known markers of endothelial cells. Markers like PLPP3, PLPP1, and EMCN further support the role of glomerular EC, where maintaining the filtration barrier is critical.
- Immune Cells. The presence of genes like PTPRC (CD45), CD4, ITGAX (CD11c), ITGAM (CD11b), and CD86 strongly suggests relationship to immune cells. PTPRC (CD45): A marker for all leukocytes. CD4: Typically associated with T helper cells, but also expressed in a subset of dendritic cells.
- LOH. UMOD (Uromodulin): Highly specific gene for LOH cells. SLC12A1 (NKCC2): A critical transporter in the LOH, involved in reabsorbing sodium, potassium, and chloride. PLCE1 and PLCB1: Involved in signal transduction, fitting the function of cells in this segment. CACNA2D3, CASR, CLCN5: Associated with ion transport, relevant to the function of LOH cells.
- Podocytes. NPHS1 (Nephrin) and PODXL (Podocalyxin) are classic markers for podocytes. WT1 (Wilms' Tumor 1) is a transcription factor highly expressed in podocytes. MAGI2 (Membrane-associated guanylate kinase) is also associated with the slit diaphragm, a key structure in podocytes. PTPRO and PTPRQ are tyrosine phosphatases involved in podocyte function. FRY and DST (Dystonin) are cytoskeletal proteins important for maintaining podocyte structure.
- Proximal Tub Cells. SLC22A8 (OAT3), SLC13A3 (NaDC3), and SLC17A1 (NPT1) are transporters commonly associated with the proximal tubule cells, which are involved in reabsorption processes in the kidney. LRP2 (megalin) and CUBN (cubilin) are also highly expressed in proximal tubule cells, where they play roles in receptor-mediated endocytosis of proteins and other molecules. SLC4A4 (NBCe1) is involved in bicarbonate transport, which is a crucial function of the proximal tubule in acid-base balance.
- Stromal cells. VIM (Vimentin) and PDGFRB (Platelet-Derived Growth Factor Receptor Beta) are classic markers of mesenchymal stromal cells, including fibroblasts, pericytes, and other supportive cells within the kidney. COL1A1 (Collagen Type I Alpha 1), TIMP1/3 (Tissue Inhibitor of Metalloproteinases), and ACTB (Actin, Beta) are involved in extracellular matrix production and remodeling, which are key functions of stromal cells. IGFBP5 (Insulin-Like Growth Factor Binding Protein 5) and AKAP12 (A-Kinase Anchoring Protein 12) are also associated with stromal cell functions, particularly in regulating cell signaling and the extracellular matrix.

Gene symbol	Gene name	Assay ID
**GAPDH**	Glyceraldehyde-3-phosphate dehydrogenase	Hs99999905_m1
**DNAJA1**	Heat shock protein family (Hsp40) member A1	Hs00266001_m1
**DNAJB6**	DnaJ heat shock protein family (Hsp40) member B6	Hs00369717_m1
**ELMSAN1**	ELM2 and SANT domain-containing protein 1	Hs00411442_m1
**HSP90AA1**	Heat shock protein 90 alpha family class A member 1	Hs05036147_s1
**HSP90AB1**	Heat shock protein 90 alpha family class B member 1	Hs01561219_m1
**HSPA4L**	Heat shock protein family A (Hsp70) member 4 like	Hs01554771_m1
**HSPA9**	Heat Shock Protein Family A (Hsp70) Member 9	Hs00269818_m1
**HSPD1**	Heat shock protein family D (Hsp60) member 1	Hs01036757_m1
**HSPH1**	Heat shock protein family H (Hsp110) member 1	Hs00971475_m1
**EIF5**	Eukaryotic translation initiation factor 5	Hs00820472_m1

Supplementary Table S2. Gene expression primers for qPCR.

Kidney no.	*Slope of eGFR(mL/min/day)*
K1	*0.90*
K2	*0*
K3	*0.19*
K4	*1.10*
K5	*0.94*
K6	*1.26*
K7	*2.29*
K8	*0.86*
K9	*0.41*
K10	*1.47*
K11	*0.32*
K12	*1.10*
K13	*0*
K14	*1.16*
K15	*0.95*
K16	*1.05*
K17	*1.15*
K18	*1.31*
K19	*16.21*
K20	*8.79*
K21	*0*
K22	*1.10*
K23	*0*
K24	*2.01*
K25	*0*
K26	*0.61*
K27	*0.38*
K28	*1.63*

Supplementary Table S3. Slopes of eGFR recovery across the early post-transplant period.
A flat or near-zero slope indicates minimal improvement over time, whereas a steeper positive slope reflects progressive graft function.

Correlation	Donor WIT	CIT1	CIT2	Total CIT	Oxygenated HMP	Donor hypertension	Donor creatine	Donor BMI	Donor weight	Urine output 60min	Urine output 120min
**DNAJA1**	R=-0.023, *P=0.918*	R=-0.256, *P=0.250*	R=0.054, *P=0.813*	R=-0.050, *P=0.826*	R=-0.002, *P=0.994*	R=0.235, *P=0.292*	R=0.333, *P=0.129*	R=0.096, *P=0.672*	R=0.126, *P=0.577*	R=-0.151, *P=0.501*	R=0.005, *P=0.982*
**DNAJB6**	R=-0.393, *P=0.070*	R=-0.185, *P=0.410*	R=0.098, *P=0.665*	R=-0.054, *P=0.813*	R=0.276, *P=0.214*	R=0.304, *P=0.169*	R=0.077, *P=0.732*	R=0.000, *P=1.000*	R=0.086, *P=0.703*	R=-0.195, *P=0.385*	R=-0.175, *P=0.437*
**ELMSAN1**	R=-0.004, *P=0.987*	R=0.135, *P=0.549*	R=0.215, *P=0.336*	R=0.296, *P=0.180*	R=0.352, *P=0.108*	R=-0.289, *P=0.192*	R=-0.333, *P=0.130*	R=0.054, *P=0.812*	R=0.010, *P=0.965*	R=-0.090, *P=0.689*	R=-0.460, *P=0.031**
**HSP90AA1**	R=-0.309, *P=0.162*	R=-0.016, *P=0.944*	R=0.086, *P=0.702*	R=0.283, *P=0.202*	R=0.284, *P=0.200*	R=0.615, *P=0.002***	R=0.102, *P=0.652*	R=-0.227, *P=0.370*	R=-0.255, *P=0.252*	R=0.070, *P=0.757*	R=0.005, *P=0.982*
**HSP90AB1**	R=0.289, *P=0.193*	R=0.219, *P=0.327*	R=0.235, *P=0.292*	R=0.201, *P=0.371*	R=0.180, *P=0.423*	R=-0.492, *P=0.020**	R=-0.448, *P=0.037**	R=0.093, *P=0.679*	R=-0.154, *P=0.495*	R=0.002, *P=0.994*	R=-0.038, *P=0.865*
**HSPA4L**	R=-0.297, *P=0.179*	R=-0.138, *P=0.539*	R=-0.139, *P=0.536*	R=-0.259, *P=0.244*	R=0.082, *P=0.718*	R=-0.051, *P=0.820*	R=-0.125, *P=0.578*	R=-0.103, *P=0.647*	R=-0.282, *P=0.204*	R=-0.072, *P=0.751*	R=0.045, *P=0.844*
**HSPA9**	R=0.086, *P=0.702*	R=-0.170, *P=0.449*	R=0.232, *P=0.299*	R=-0.054, *P=0.812*	R=-0.109, *P=0.629*	R=0.049, *P=0.830*	R=0.086, *P=0.763*	R=-0.069, *P=0.761*	R=-0.135, *P=0.549*	R=-0.406, *P=0.061*	R=-0.186, *P=0.406*
**HSPD1**	R=-0.094, *P=0.677*	R=-0.040, *P=0.861*	R=0.187, *P=0.405*	R=0.051, *P=0.823*	R=0.246, *P=0.270*	R=-0.510, *P=0.015**	R=-0.419, *P=0.052*	R=0.033, *P=0.884*	R=-0.118, *P=0.602*	R=-0.170, *P=0.451*	R=-0.235, *P=0.293*
**HSPH1**	R=-0.041, *P=0.855*	R=0.003, *P=0.988*	R=-0.055, *P=0.809*	R=0.030, *P=0.894*	R=0.199, *P=0.376*	R=0.266, *P=0.231*	R=0.046, *P=0.838*	R=-0.040, *P=0.859*	R=-0.100, *P=0.656*	R=-0.270, *P=0.225*	R=-0.306, *P=0.167*
**EIF5**	R=0.159, *P=0.480*	R=0.152, *P=0.500*	R=0.298, *P=0.179*	R=0.307, *P=0.164*	R=-0.099, *P=0.660*	R=-0.225, *P=0.134*	R=-0.059, *P=0.795*	R=0.274, *P=0.218*	R=0.109, *P=0.628*	R=-0.162, *P=0.472*	R=-0.072, *P=0.749*

Supplementary Table S4. Correlation analyses between gene expression changes induced by two-hour NMP and clinical outcomes.
*. Correlation is significant at the 0.05 level (two-tailed); **. Correlation is significant at the 0.01 level (two-tailed).
